# A Case of Fracture-Redislocation of the Hip Caused by a Depressed Fracture of the Femoral Head Similar to a Hill-Sachs Lesion

**DOI:** 10.1155/2017/7409153

**Published:** 2017-05-18

**Authors:** Yoshihiko Okudera, Hiroaki Kijima, Shin Yamada, Natsuo Konishi, Hitoshi Kubota, Hiroshi Tazawa, Takayuki Tani, Norio Suzuki, Keiji Kamo, Ken Sasaki, Tetsuya Kawano, Yosuke Iwamoto, Naohisa Miyakoshi, Yoichi Shimada

**Affiliations:** ^1^Akita Hip Research Group (AHRG), Akita, Japan; ^2^Department of Orthopedic Surgery, Akita University Graduate School of Medicine, Akita, Japan

## Abstract

The present case shows a case of fracture-redislocation of the hip caused by a depressed fracture of the femoral head similar to a Hill-Sachs lesion. A 59-year-old man fell from a roof and his left hip joint was dislocated posteriorly. He was admitted to a nearby hospital, and he was referred to our hospital more than 24 hours after injury. Computed tomography (CT) suggested a bone chip from the posterior wall of the acetabulum roof and a depressed femoral head that cut into the posterior margin of the acetabulum roof. Immediate manual repositioning was performed under general anesthesia on the same day. He left our hospital to go home on day 26 after repositioning, but his left hip joint was dislocated again when he went down the stairs. It was thought that this patient's redislocation occurred due to a femoral head depressed fracture involving the same mechanism as the Hill-Sachs injury seen with dislocation of the shoulder. The remplissage method for the Hill-Sachs injury is difficult for the femoral head. Therefore, total hip replacement was performed, and the patient's postoperative course was good. We conclude that total hip arthroplasty should be considered as one of the best treatment methods for such cases.

## 1. Introduction

The Pipkin classification is one of the best-known classifications of fractures of the femoral head, and it may be of use in determining treatment strategy. A case of fracture-dislocation of the hip, in which a depressed fracture of the femoral head that was unclassifiable by this classification caused easy redislocation and required treatment, is reported.

## 2. Case Presentation

A 59-year-old man presented with a chief complaint of pain in the left hip. His previous medical history included nothing of note.

### 2.1. History of Current Condition

The patient had injured himself by falling from a roof, and he had been admitted to a local clinic on the same day. He was referred to our department after more than 24 hours had elapsed since the injury occurred.

### 2.2. Findings on Initial Examination

**Figure 1:**
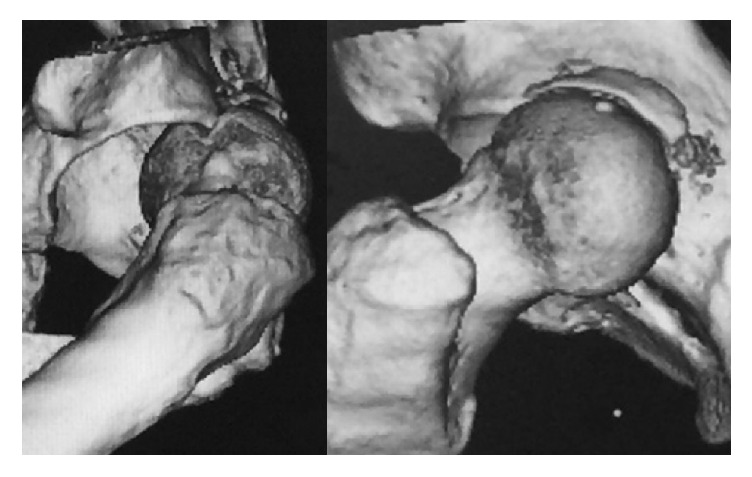
Computed tomography suggested a bone chip from the posterior wall of the acetabular roof and a depressed femoral head that cut into the posterior margin of the acetabular roof.

The left hip was immobilized at 40° flexion. Plain X-ray revealed posterior dislocation of the hip, and, on CT, not only was a posterior wall bone fragment measuring 24 mm × 10 mm × 3 mm visible, but the posterior margin of the acetabulum had created a depression in the femoral head into which it was impacted (Figure 1).

### 2.3. Course

**Figure 2:**
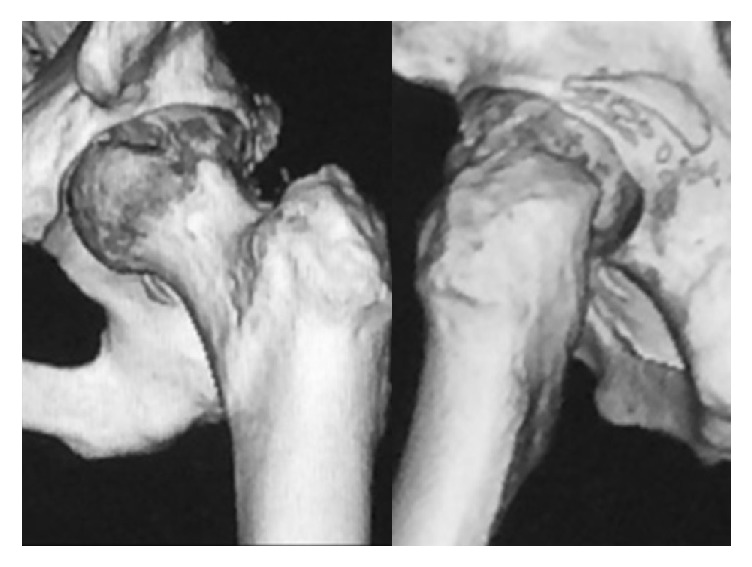
Computed tomography of the left hip joint after reduction.

**Figure 3:**
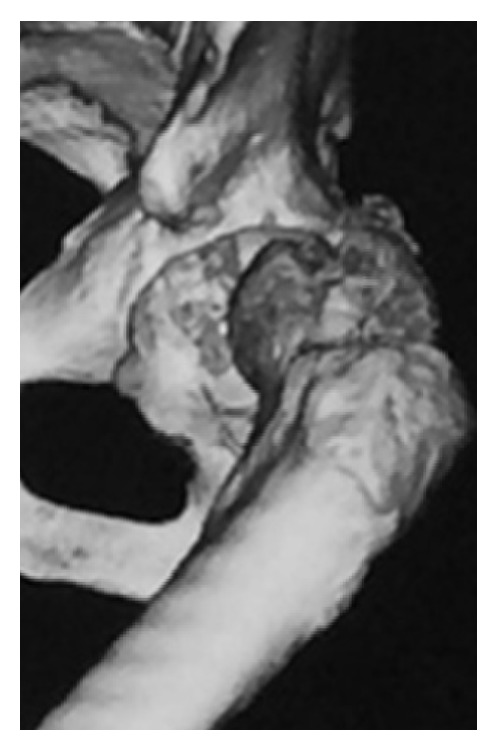
Computed tomography of the left hip joint after redislocation.

**Figure 4:**
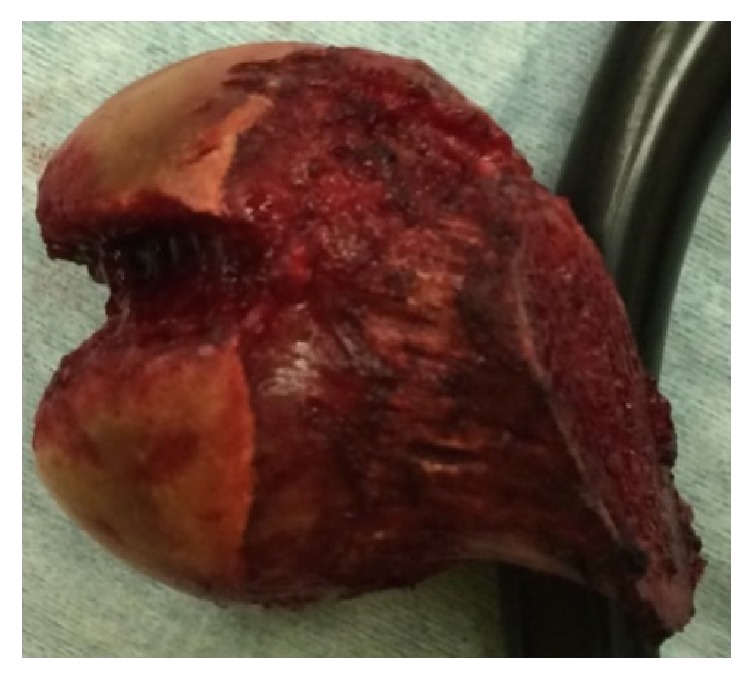
Removed femoral head. The depressed fracture in the femoral head is observed.

**Figure 5:**
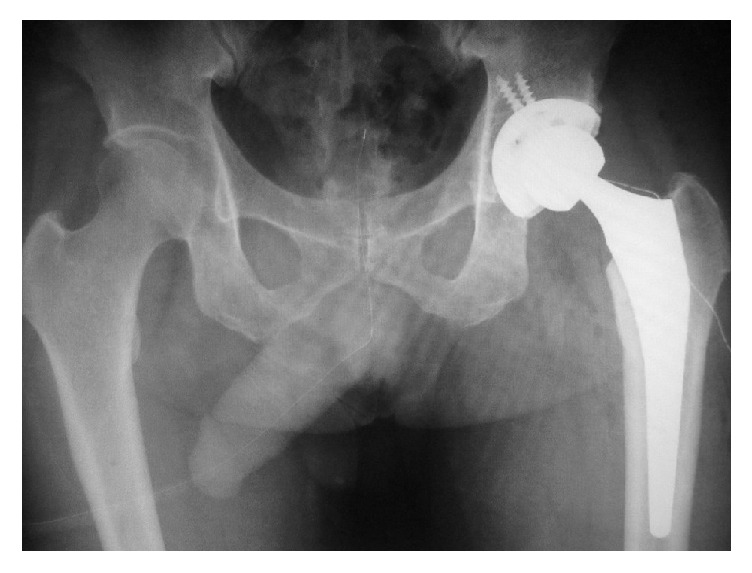
Radiographs of the hip joint after total hip arthroplasty.

Emergency manual reduction was performed on the same day under general anesthesia (Figure 2). The patient was fitted with an abduction pillow and was permitted to move around in a wheelchair from day 15 after manual reduction, but since he was not in pain, he started walking without permission. He was discharged home on day 26, but 4 days after discharge, when he was climbing down a step with his right leg, his left hip again became dislocated posteriorly in a manner similar to the initial injury, and he was readmitted to hospital (Figure 3). Manual reduction was again attempted, but because the hip dislocated easily, total hip arthroplasty was performed (Figures 4 and 5), and the patient's subsequent course has been uneventful even 1 year after the surgery.

## 3. Discussion

The Pipkin classification is one of the best-known classifications of fractures of the femoral head [1], and although this does not set out clear surgical indications [2], it is frequently useful for determining a treatment strategy. In the present case, the dislocation took the form of a depression in the femoral head caused by the posterior wall of the acetabulum, a state unclassifiable according to Pipkin's classification.

There are no set criteria for the use of surgery to treat fractures of the posterior wall of the acetabulum, although one study has reported that surgery should be performed if the posterior wall bone fragment comprises more than 1/3 of the joint surface [3]. In this case, the posterior wall bone fragment was small, and after the initial dislocation was reduced, a conservative approach was adopted. However, redislocation occurred.

It is unlikely that the extremely small bone fragment of the posterior wall of the acetabulum could have caused this redislocation by itself. In this case, CT prior to the reduction showed that the depressed fracture in the femoral head had caused the dislocation by the same mechanism whereby a Hill-Sachs lesion causes shoulder dislocation [4]. In shoulder dislocations, however, the cause of redislocation is normally a lesion in the inferior glenohumeral ligament complex (Bankart lesion), and it is rare for a Hill-Sachs lesion to cause redislocation. Nevertheless, in cases in which the Hill-Sachs lesion crosses the glenoid track of the humeral head, the indentation of the Hill-Sachs lesion may become trapped in the glenoid margin, causing redislocation [5]. It was this mechanism that may have caused the redislocation in the present case.

Depressed fractures of the femoral head have reportedly been treated conservatively if dislocation has not occurred [6], but as far as we have been able to ascertain from a survey of the literature, no case has been treated with surgery. In the present case, however, the depressed fracture of the femoral head was causing redislocation, and some sort of invasive treatment was therefore required.

We should consider valgus/rotational proximal femur osteotomy. However, the patient was regarded as at high risk of femoral head necrosis and the patient needs the long period of non-weight-bearing if the osteotomy is performed. Thus, in this case we suggested total hip arthroplasty for the patient.

One surgical technique associated with the mechanism of onset is the use of remplissage to treat Hill-Sachs lesions [7]. The soft tissue in the hip that corresponds to the rotator cuff in the shoulder is the gluteus medius tendon, but there has been no report of the use of the gluteus medius tendon in a procedure similar to remplissage, and even if it were to be performed it would be expected to result in severely restricted postoperative range of motion, requiring a long period of rehabilitation. Therefore, total hip arthroplasty was performed in the present case with a good outcome. Since the reduction was performed more than 12 hours after the initial dislocation, the patient was regarded as at high risk of femoral head necrosis [8], and, taking this into consideration, total hip arthroplasty should be considered as one option in similar cases.

## 4. Conclusion

When a depressed fracture of the femoral head is present in a fracture-dislocation of the hip, this may cause redislocation even if the bone fragment of the posterior wall of the acetabulum is small.
